# Insomnia management in Dutch general practice: a routine care database study

**DOI:** 10.1080/02813432.2023.2237073

**Published:** 2023-07-20

**Authors:** Mette H. Bakker, Nina A. Oldejans, Jacqueline G. Hugtenburg, Henriëtte E. van der Horst, Pauline Slottje

**Affiliations:** a Department of General Practice, Amsterdam UMC, Vrije Universiteit Amsterdam, Amsterdam, the Netherlands; b Amsterdam Public Health Research Institute, Quality of Care, Amsterdam, the Netherlands; c Department of Clinical Pharmacology and Pharmacy, Amsterdam UMC, Vrije Universiteit Amsterdam, Amsterdam, the Netherlands

**Keywords:** Insomnia, sleep disturbance, general practice, hypnotics, sleep medication

## Abstract

**Objective:**

To explore insomnia management in general practice, with a focus on sleep medication prescription.

**Design:**

Descriptive analysis of anonymized routine general practice care data extracted from electronic medical records (EMRs), including demographics, free text annotations from sleep consultations and sleep medication prescriptions covering one year before up to two years after the registration of the International Classification for Primary Care (ICPC) code P06 ‘Sleep disturbance’.

**Setting:**

Twenty-one general practices in an urban area of the Netherlands.

**Patients:**

Adults (18–85 year) with a first sleep consultation with their GP.

**Outcomes:**

Documented non-pharmacological and sleep medication treatment.

**Results:**

Of the 1,089 patients who consulted their general practitioner (GP) for sleep disturbance for the first time, about 50% had one more sleep consultation during the two years follow-up. Over two years including the first consultation, GPs documented a non-pharmacological intervention for 48.4% of the patients and prescribed sleep medication to 77.0%. 64.6% of the patients received a sleep medication prescription in the first consultation. Among patients receiving medication (*N* = 838); 59.6% received more than one prescription; 76.8% received one or more short-acting benzodiazepine receptor agonist (BZRA), 39.5% one or more unrecommended drugs and 14.7% >180 pills of BZRAs in two years.

**Conclusion:**

Although the guidelines advocate non-pharmacological treatment and warn against unwarranted sleep medication, it is still very common in Dutch general practice to prescribe medication, even at the first sleep consultation. Prescriptions frequently include unrecommended and off-label drugs or repeated BZRA prescriptions.

## Key points

Short statement

There are concerns about the prescription rate and nature of sleep medication prescribed for patients with sleep disturbance. Analysis of routine care data can provide insights in general practitioners (GPs) management of insomnia.

**3 main statements**

1. GPs prescribed sleep medication in the first consultation to 64.6% of the patients who consulted them for sleep disturbance for the first time.
2. Over two years including the first consultation, 48.4% of the patients received a (documented) non-pharmacological intervention, 77.0% a sleep medication prescription.
3. Among patients receiving medication, the majority received short-acting benzodiazepine receptor agonists (BZRAs), but 39.5% (also) received an unrecommended drug.

## Introduction

Insomnia is the most prevalent sleep disorder. Worldwide about 30 to 48 percent of the adult population suffers from one or more insomnia symptoms such as difficulties initiating or maintaining sleep, or non-restorative sleep [1]. Nine to 15 percent of the adult population fulfills the criteria for insomnia diagnosis, which is defined as sleep difficulties accompanied by daytime consequences, sometime with additional criteria for the frequency or duration of the complaints [1,2]. In about six percent of the adult population insomnia last for three months or longer and the diagnostic criteria for insomnia disorder are met [1,3]. Insomnia is associated with lower quality of life and development of for instance cardiovascular and mental disorders [4–6]. Insomnia contributes to a decreased work productivity and increased health care costs [7,8]. In countries such as the Netherlands, the United Kingdom, Norway, Sweden, Finland, Canada and Australia, insomnia management largely takes place in primary care. Patient commonly visit their doctor when the insomnia symptoms affect their functioning [9].

There are two ongoing concerns about insomnia management. First, sleep medication is overprescribed, while effective non-pharmacological treatments are insufficiently applied [6,10,11]. Secondly, the health risks of both the licensed and off-label prescribed drugs may outweigh their efficacy. International guidelines advocate non-pharmacological treatments and advise to limit sleep medication prescriptions [2,12–15]. In practice however, effective strategies such as stimulus control, sleep restriction and cognitive behavioral therapy for insomnia (CBT-I) are barely applied or insufficiently available [16–18]. Benzodiazepine receptor agonist (BZRA) sleep medication (i.e. benzodiazepines and z-drugs) are often prescribed for longer periods than recommended, thus raising the risk of harmful side effects and dependence [14,19,20]. Off-label drugs are increasingly prescribed as an alternative for BZRAs, but expose patients to health risks without proven benefit [21,22].

Interview and survey studies among GPs have identified factors associated with the decision whether or not to prescribe sleep medication [23–29]. These included availability of treatment, GPs’ experiences and preferences as well as patient factors such as insomnia severity and duration and comorbidity such as depression and anxiety. These studies, however, lack quantified care data on the occurrence of such prescriptions. Prescription database studies, on the other hand, often consider only BZRA sleep medication and miss contextual information [30].

The aim of this study was to provide descriptive epidemiological data on general practitioners (GPs) insomnia management in patients who consulted their general practitioner (GP) for sleep disturbance for the first time, with a focus on sleep medication prescription. Data at patient level from electronic medical records covering one year before up to two years after the registration of the International Classification for Primary Care (ICPC) code P06 ‘Sleep disturbance’ were hereby used.

The following research questions are addressed:

1. How do GPs manage patients with sleep disturbance from the first sleep consultation onwards to the following two years: how often are non-pharmacological interventions recorded, how often do they advise over-the-counter (OTC) remedies? How often do they prescribe sleep medication?
2. Which percentage of patients does receive a prescription for sleep medication during the first sleep consultation and during the first two years following this consultation?
3. What is the nature and frequency of sleep medication (recommended/unrecommended) prescribed; and in what order drugs are prescribed?
4. Do patient and sleep characteristics differ for patients who received one or more prescriptions for sleep medications compared to patients who did not receive any prescription?

## Methods

### Study design

This observational study was carried out with anonymized longitudinal routine general practice care data extracted from the database of the Academic Network of General Practice at VU University Medical Center (ANH VUmc), Amsterdam. We used the anonymized data extracted from the electronic medical records (EMR) of 75,720 enlisted adult patients in 21 general practices in the city of Amsterdam in the five-years period between 1 January 2013 and 31 December 2017.

### Study population

Our target population consisted of adult patients who consulted their GP for sleep disturbance for the first time, i.e. the GP recorded upon consultation the International Classification for Primary Care (ICPC) code P06 ‘Sleep disturbance’ for the first time in the patients EMR (i.e. the first sleep consultation). This was excluding obstructive sleep apnea syndrome (OSAS), i.e. subcode P06.01. We assumed that the vast majority of them had insomnia, since it has been estimated that insomnia accounts for 90% of the consultations for sleep disturbance in general practice [2]. We applied the following criteria in the search algorithm to select our study population: First, all enlisted patients aged >18 years at 31 December 2017 with a P06 episode (at a given index date) after the publication date of the current Dutch insomnia guideline (1 July 2014) [2] were selected. Secondly, patients without EMR data from one year before to two years after the index date, were excluded. This assured that the selected patients consulted their GP for sleep disturbance for the first time, as far as could be traced back in the patients EMR available for the GP with a minimum period of one year. Thirdly, patients aged <18 or ≥83 years (i.e. children and the eldest elderly above 85 at end of follow up) at the index date were excluded. We decided to focus on the middle age groups. This was because of pragmatic reasons and because the context of first consultation for sleep disturbance in the youngest and oldest groups might be clinically different from the rest, possibly requiring a different search strategy. Lastly, patients were excluded in case the free text annotations of the P06 contact(s) showed that they had another problem than insomnia (e.g. patients who had another specific sleep disorder such as narcolepsy) or anticipated sleep disturbance rather than present sleep disturbance (e.g. patients who had a sleep medication request because of planned traveling). Obvious miscodings were also excluded.

### Dutch insomnia guideline

We explored insomnia management by GPs in the context of the current Dutch insomnia guideline, published in 2014 [2]. In this guideline, insomnia is defined as sleep difficulties for at least three times a week, accompanied by daytime consequences, such as impaired functioning, fatigue, sleepiness, irritability, reduced concentration and performance. For insomnia it recommends non-pharmacological interventions. This should include education about normal sleep and, in cases insomnia persists for three weeks or longer, a behaviural approach in which ‘sleep hygiene’ advises (i.e. healthy sleep habits), stimulus control, sleep restriction, relaxation exercises, cognitive therapy and physical exercises are combined. It advises to only prescribe sleep medication for a short term (a short-acting BZRA up to 10 tablets) in exceptional situations: i.e. (1) as temporarily relief when the sleep disturbance is of recent origin and has an identifiable cause of transitory nature or (2) as temporarily relief when the sleep disturbance is chronic and leads to dysfunctions in daily life, and improvement is not otherwise expected.

### Data extraction and categorization

For the selected patients consultation data, prescription data, and background data were extracted covering the period between the year before and the two years after the index date of the first sleep disturbance episode. The extracted data were systematically reviewed and categorized by three instructed researchers (a GP (MB), a medical student (NO) and a research assistant (social scientist)) using a protocol consisting of scoring rules for free-text variables and scoring rules to determine whether a drug is prescribed as sleep medication or not. To increase the reliability a randomly selected 10% of the patients were reviewed by two researchers (MB and NO) independently. All doubts were discussed until consensus was reached.

#### Consultation data

Contact type, date and free-text annotation data were retrieved from home visits, consultations at the general practice, phone calls and email contacts classified by the GP with the International Classification of Primary Care (ICPC) P06 in the two years following the first P06 contact (i.e. first consultation). For each patient we calculated the number of such ‘sleep consultations’. From the free-text data of these sleep consultations we deduced: (1) duration of the sleep disturbance at the first consultation, (2) the presence of an exceptional situation in which sleep medication could be prescribed according to the national guideline, (3) the provision of non-pharmacological interventions and (4) the advice to use over-the-counter (OTC) remedies (definitions and categorization listed in Table 2). Annotated sentences were scored as non-pharmacological interventions when they suggested education, advises or support provided by the GP (e.g. ‘explanation about why no sleep medication'’, ‘sleep advises, ‘conversation'’, which is a common phrase for supportive chat) or when the GP annotated a referral or conversation about a non-pharmacological intervention (e.g. ‘referral to [psychologist/yoga etc]’, ‘alternatives to sleep medication offered’). Annotations meant to document advice to limit the use of sleep medication (e.g. ‘use sleep medication only as an escape’) and annotations deferring further policy (e.g. ‘wait and see'’, ‘referral to Sleep Clinic for further diagnostics’) were not scored as non-pharmacological interventions.

#### Sleep medication prescriptions

Prescription data consisted of the Anatomical Therapeutic Chemical (ATC) classification of medicines potentially prescribed as sleep medication (listed in Table 1), prescription date and free text data associated with the prescription (e.g. dosage). Table 1 presents the scoring rules used to determine whether a drug was prescribed as sleep medication or not. Per patient we calculated the number of prescriptions over two years (0, 1 or >1) and number of drugs prescribed in two years (0, 1 2 or 3 or more). Sleep medication was subsequently further categorized. First, we distinguished BRZA (ATC codes N05BA, N05CD, N05CF) from non-BRZA (all other prescribed sleep medication). Secondly, prescribed drugs were categorized according to the national guideline into ‘recommended’ (short-acting BZRAs), ‘licenced sleep medication but not recommended in the national guideline’ (other BZRA sleep medication and melatonin) and ‘not licenced sleep medication and unrecommended’ in the national guideline (off-label drugs) [2].

**Table 1.** Scoring rules to determine whether a drug is prescribed as sleep medication or not.

1. A drug^a^ is sleep medication when it pertains:
Temazepam, zolpidem, zopiclone, lormetazepam, melatonin
*and/or*
Explicitly documented as sleep medication in free-text of the consultation on the same day
*and/or*
‘For sleep‘ documented in prescription-texts
2. A drug is no sleep medication when
Another indication is more likely, based on
-Another ICPC code than P06 assigned to prescription
-Documentation in free-text or prescription text referring to another indication
-Another drug which is considered sleep medication (according to step 1.) is prescribed on the same day
-Prescribed several times a day
-Unlikely dosages for sleep medication^b^
*or*
There is lack of information on the indication for this prescription

^a^ATC codes of potential sleep medication: N05BA, N05CD, N05CF (BRZA; benzodiazepines and z-drugs), N05CH (melatonin), N05AH, N05AD (antipsychotics), N06AA, N06AX (antidepressants), N03AX, N03AE01 (antiepileptics), R06AD02, R06AD01, R06AA04, R06AB03, R06AX17, R06AD08, N05BB01 (antihistamines).

^b^Mirtazapine ≥30 mg, amitriptyline ≥50 mg, quetiapine ≥100mg, olanzapine ≥10 mg, clonazepam ≥2 mg.

In addition, to explore treatment order, prescribed drugs were ordered based on their first prescription date into first drugs, second drugs and subsequent (third to seventh) drugs. Finally, long term BZRA prescription was defined as a total sum of all BRZA pills prescribed above 180 in two years, which is a commonly used cut off points for long term usage [31]. In rare cases of two sleep medication prescriptions of the same drug on one day, the one with the highest number of pills was taken into account.

#### Background data

Background data included age, sex and psychological problems and social problems. Concurrent psychological problems (i.e. psychological symptoms or diagnosis) or social problems (e.g. financial, housing, or relationship problems) were defined as an episode coded with ICPC-codes in the ‘P‘ or ‘Z‘ domain respectively with a starting date in the year before or in the two years after the first sleep consultation.

### Data analysis

We used descriptive statistics to describe background characteristics and the insomnia management in the total study population and stratified by whether or not sleep medication was prescribed. Numbers and percentages or mean and standard deviation are presented. We described type and order of sleep medication prescription in the population receiving at least one prescription. We performed univariate logistic regression analyses to explore the association between patient characteristics and the prescription of sleep medication in two years of follow-up time. Age was analyzed as a continuous variable per five years. Sex, concurrent psychological problems and socials problems were dichotomous. Thereafter, as we found a significant association for age, we analyzed the relation between sex, concurrent psychological problems and social problems and sleep medication prescription corrected for age. We presented odds ratios (ORs) with a 95 percent confidence interval (CI). We waived the intended explorations on associations between sleep characteristics and sleep medication prescription, as in the majority of the patients (74.6%) the GP had not documented the duration of the sleep disturbance at the first consultation. Since we aimed to describe routine care and half of the patients were found to have only one sleep consultation (i.e. the first consultation) over two years, we conducted a post hoc analysis. In this univariate logistic regression analysis we stratified by 1 sleep consultation versus >1 sleep consultation to determine whether this affected medication prescribing and the provision of documented non-pharmacological care. Data were analyzed using SPSS version 28.0.

## Results

### Patient selection

**Figure 1.**
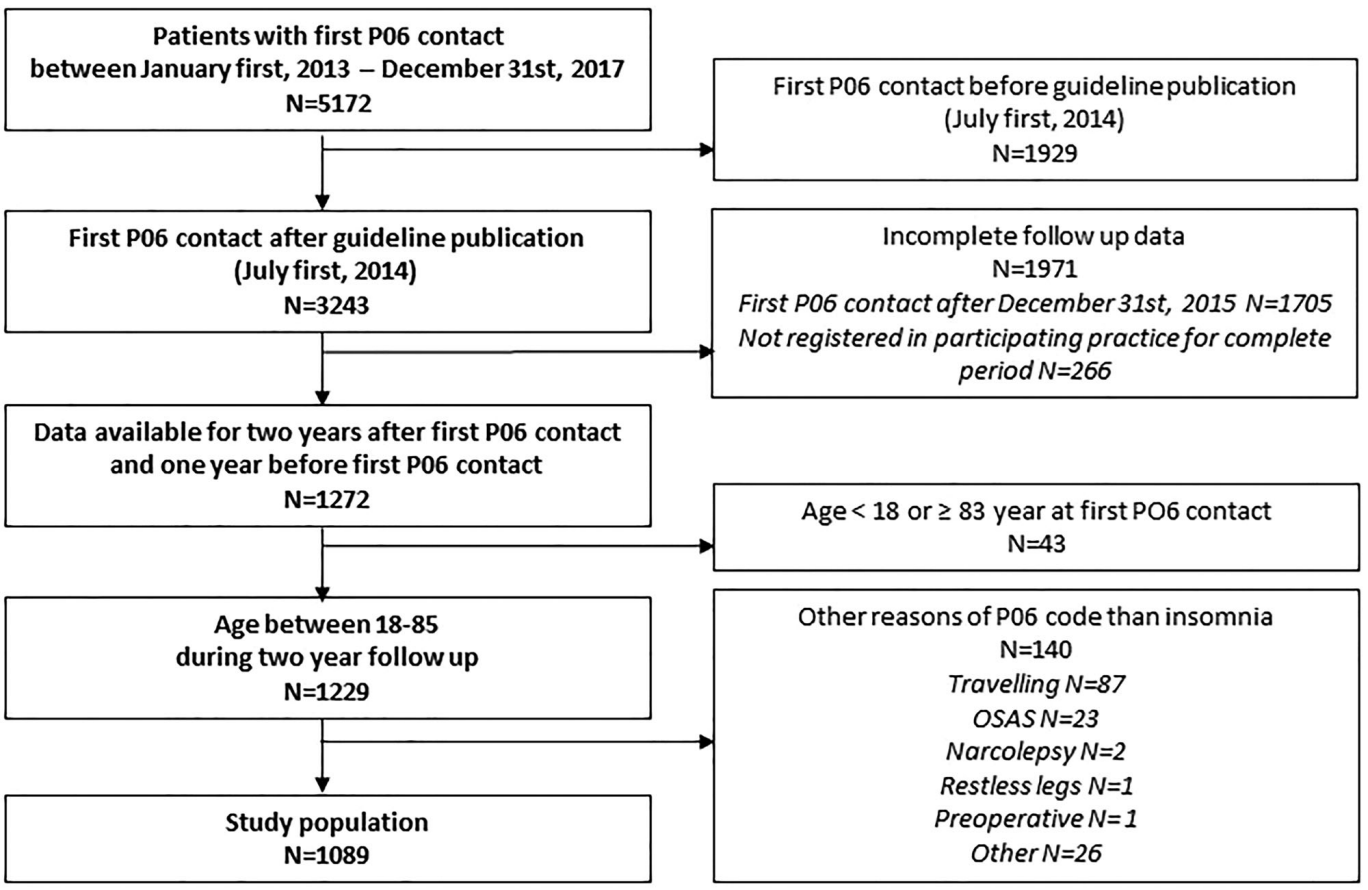
Patient selection flow chart. P06: International Classification of Primary Care (ICPC) P06 code ‘Sleep disturbance’ ‘assigned to a contact by the GP. OSAS: obstructive sleep apnea syndrome; this concerned cases that were incorrectly coded as P06 instead of P06.01.

Figure 1 shows the patient selection flow chart; 1089 patients, who consulted their GP for sleep disturbance for the first time, met the inclusion criteria of the study.

### Patient and sleep characteristics

Table 2 shows background characteristics and the GP’s insomnia management over two years including the first sleep consultation in the total study population and stratified by whether or not sleep medication was prescribed. The duration of the sleep disturbance at the first consultation was annotated in 32.8% of the patients. In 8.4% (*N* = 91) of the patients the presence of a crisis (i.e. acute (worsened) sleep disturbance with an identifiable cause of transitory nature as defined in the national guideline) was annotated; 81 of them received one or more sleep medication prescription, 10 did not. Patients had on average 2.6 (SD 2.8) sleep consultations with their GP in two years; 74.4% of the consultations took place at the practice, 23.3% were phone consultations, 1.6% home visits and 0.7% E-consultations. Approximately half of the patients (49.8%) had only one sleep consultation in two years.

**Table 2.** Background characteristics and general practitioner’s insomnia management in total study population and stratified by whether or not sleep medication was prescribed.

	Total study population, patients with first sleep consultation N = 1089	No sleep medication prescribed 251 (23.0%)	Sleep medication prescribed 838 (77.0%)
**Patient characteristics**			
Age at first consultation			
Mean in years (SD)	48.8 (SD 16.1)	45.0 (SD 16.7)	49.9 (SD 15.8)
18–25 years	76 (7.0%)	34 (13.5%)	42 (5.0%)
26–40 years	295 (27.1%)	73 (29.1%)	222 (26.5%)
41–65 years	503 (46.2%)	99 (39.4%)	404 (48.2%)
66–83 years	215 (19.7%)	45 (17.9%)	170 (20.3%)
Sex			
Female	699 (64.2%)	163 (64.9%)	536 (64.0%)
Concurrent psychosocial problem^a^			
Any concurrent psychological problem	523 (48.0%)	110 (43.8%)	413 (49.3%)
*Depressive disorder diagnosis*	*76 (7.0%)*	16 (6.4%)	60 (7.2%)
*Anxiety disorder diagnosis*	*89 (8.2%)*	22 (8.8%)	67 (8.0%)
Any concurrent social problem	239 (21.9%)	55 (21.9%)	184 (21.9%)
**Sleep characteristics**			
Duration of sleep disturbance at first consultation^b^			
<3 weeks	99 (9.1%)	19 (7.6%)	80 (9.5%)
3 weeks–3 months	39 (3.6%)	13 (5.2%)	26 (3.1%)
≥3 months	139 (12.8%)	47 (18.7%)	92 (11.0%)
Not (clearly) annotated	812 (74.6%)	172 (68.5%)	640 (76.4%)
**Sleep consultations**			
Number of sleep consultations^c^ in two years, including first consultation			
Number of consultations, mean (SD)	2.6 (SD 2.8)	1.7 (SD 1.7)	2.8 (SD 3.0)
>1 consultation	547 (50.2%)	82 (32.7%)	465 (55.5%)
**Insomnia treatment by GP**			
Documented non-pharmacological intervention (NPI)^d^			
Any documented NPI	527 (48.4%)	162 (64.5%)	365 (43.6%)
*Only NPI*	*162 (14.9%)*	*162 (64.5%)*	*n.a.*
*NPI before sleep medication*	*53 (4.9%)*	*n.a.*	*53 (6.3%)*
*NPI and sleep medication at the same time*	*222 (20.4%)*	*n.a.*	*222 (26.5%)*
*NPI after sleep medication*	*90 (8.3%)*	*n.a.*	*90 (10.7%)*
Not annotated	562 (51.6%)	89 (35.5%)	473 (56.4%)
Documented advise over-the-counter (OTC) remedies^e^			
Any documented OTC advise	69 (6.3%)	35 (13.9%)	34 (4.1%)
*Melatonin OTC advised*	*48 (4.4%)*	*25 (10.0%)*	*23 (2.7%)*
*Valerian OTC advised*	*16 (1.5%)*	*7 (2.8%)*	*9 (1.1%)*
*Other OTC remedy advised*	*12 (1.1%)*	*5 (2.0%)*	*7 (0.8%)*
Not annotated	1020 (93.7%)	216 (86.1%)	804 (95.9%)
Prescribed sleep medication			
No sleep medication	251 (23.0%)	251 (100.0%)	n.a.
1 prescription of single drug	340 (31.2%)	n.a.	340 (40.6%)
>1 prescriptions of 1 (the same) drug	307 (28.2%)	n.a.	307 (36.6%)
>1 prescriptions, 2 different drugs	140 (12.9%)	n.a.	140 (16.7%)
>1 prescriptions, 3–7 different drugs	51 (4.7%)	n.a.	51 (6.1%)

*Note:* Table presents numbers (%) of patients, covering insomnia management over two years including the first sleep consultation i.e. unless stated otherwise.

Abbreviations: GP: general practitioner; EMR: electronical medical record; NPI: non-pharmacological intervention; OTC: over-the-counter remedies.

Subcategories are presented in italics.

^a^Concurrent psychological problem and social problem defined as an episode coded with ICPC-codes in the ‘P‘ or ‘Z‘ domain respectively with a starting date between the year before and the two years after the first consultation.

^b^Explicit documentation of the duration in the free-text annotations of the first sleep consultation and categorized according to the national guideline into <3 weeks, ≥3 weeks, but <3 months, or ≥3 months.

^c^Defined as a home visit consultation, consultation at the general practice, phone or email consultation where the GP assigned ICPC-code ‘P06‘ to.

^d^Documentation of e.g. sleep education, conversation, yoga, sleep hygiene, referral to a psychologist in the free-text annotation of at least one of the sleep consultations.

^e^Explicit advise of the GP to buy or use a specific over-the-counter remedy in the free-text annotations of at least one of the sleep consultations.

### Non-pharmacological interventions and sleep medication prescription

Over two years including the first consultation, the provision of a non-pharmacological intervention was documented in the EMR of 48.4% of the patients; in 64.5% of the patients receiving no sleep medication prescription and in 43.6% of the patients receiving sleep medication. In 19.8% of the total study population the documentation of the non-pharmacological intervention preceded sleep medication prescription or was the only treatment. The advice to buy OTC remedies was annotated in 6.3% of the patients. In the total study population 77.0% (*N* = 838) received one or more prescriptions for sleep medication. 64.6% of the total study population received a prescription for sleep medication in the first consultation (56.0% a BZRA, 8.6% non-BRZA).

### Frequency, nature and order of prescribed sleep medication

Of 838 patients who received one or more prescription, 40.6% received one single prescription, 36.6% repeat prescriptions of one drug and 16.7% multiple prescriptions of two drugs and 6.1% multiple prescriptions of three to seven drugs over two years including the first sleep consultation (Table 2).

In this group receiving medication, 76.8% received one or more short-acting BRZA as recommended in the national guideline, 39.5% received one or more other drugs not recommended by the guideline such as one or more other BZRA licensed for sleep (22.1%), melatonin (10.5%) or one or more off-label drugs (12.2%). In total 27 different drugs were prescribed as sleep medication by the GPs (Table 3). The most commonly prescribed drugs not licensed for sleep were the antidepressants mirtazapine (6.1%) and amitriptyline (2.0%). Off-label drugs further included antihistamines, antipsychotics, anxiolytics and antiepileptics. The proportion of patients receiving prescriptions for off-label drugs was 6.3% among those patients receiving a first drug. As second and subsequent drugs the proportion of off-label drugs increased to 18.3% and 35.3% respectively. 14.7% received long term BZRA prescriptions (>180 pills) in two years.

**Table 3.** Number and percentage of patients receiving at least one prescription of a drug, percentage of patients receiving > 1 prescription per drug over two years including the first sleep consultation in general practice, number and percentage of patient receiving a prescription for a drug as first drug, second drug and subsequent drug.

Prescribed drug or drug category	Number of patients receiving ≥1 prescription in two years *N* = 838 patientss	% of patients receiving >1 prescription of a drug per drug	Number of patients receiving ≥1 prescription as *first* drug *N* = 838	Number of patients receiving ≥1 prescription as *second* drug *N* = 191	Number of patients receiving ≥1 prescription as *subsequent* (third to seventh) drug *N* = 51
Licensed sleep medication, recommended in national guideline					
≥1 Short-acting BZRA	664 (76.8%)		598 (71.4%)	93 (48.7%)	23 (45.1%)
*Temazepam*	*457 (54.5%)*	*46.6%*	*407 (48.6%)*	*43 (22.5%)*	*7 (13.7%)*
*Zolpidem*	*133 (15.9%)*	*50.4%*	*111 (13.2%)*	*15 (7.9%)*	*7 (13.7%)*
*Zopiclon*	*110 (13.1%)*	*56.4%*	*74 (8.8%)*	*28 (14.7%)*	*8 (15.7%)*
* Lormetazepam*	*18 (2.1%)*	*72.2%*	*8 (1.0%)*	*7 (3.7%)*	*3 (5.9%)*
Licensed sleep medication, but not recommended in national guideline					
≥1 Other BZRA	185 (22.1%)		125 (14.9%)	58 (30.4%)	18 (35.3%)
* Oxazepam*	*87 (10.4%)*	*60.9%*	*58 (6.9%)*	*24 (12.6%)*	*5 (9.8%)*
*Lorazepam*	*31 (3.7%)*	*51.6%*	*18 (2.1%)*	*8 (4.2%)*	*5 (9.8%)*
*Diazepam*	*31 (3.7%)*	*54.8%*	*18 (2.1%)*	*7 (3.7%)*	*6 (11.8%)*
* Nitrazepam*	*27 (3.2%)*	*55.6%*	*13 (1.6%)*	*9 (4.7%)*	*5 (9.8%)*
*Midazolam*	*9 (1.1%)*	*33.3%*	*6 (0.7%)*	*2 (1.0%)*	*1 (2.0%)*
*Brotizolam*	*8 (1.0%)*	*62.5%*	*6 (0.7%)*	*2 (1.0%)*	*0 (0.0%)*
* Other^a^*	*13 (1.6%)*	*76.9%*	*6 (0.7%)*	*6 (3.1%)*	*1 (2.0%)*
Licensed sleep medication, but not recommended in national guideline					
≥1 Melatonin	88 (10.5%)		75 (8.9%)	9 (4.7%)	4 (7.8%)
* Melatonin*	*88 (10.5%)*	*21.6%*	*75 (8.9%)*	*9 (4.7%)*	*4 (7.8%)*
Not licensed sleep medication and unrecommended in national guideline					
≥1 Off-label	102 (12.2%)		53 (6.3%)	35 (18.3%)	18 (35.3%)
*Mirtazapine*	*51 (6.1%)*	*66.7%*	*21 (2.5%)*	*21 (11.0%)*	*9 (17.6%)*
*Amitriptyline*	*17 (2.0%)*	*70.6%*	*9 (1.1%)*	*6 (3.1%)*	*2 (3.9%)*
* Promethazine*	*12 (1.4%)*	*75.0%*	*5 (0.6%)*	*5 (2.6%)*	*2 (3.9%)*
*Quetiapine*	*8 (1.0%)*	*100.0%*	*3 (0.4%)*	*1 (0.5%)*	*4 (7.8%)*
* Other* ^b^	*22 (2.6%)*	*59.1%*	*15 (1.8%)*	*2 (1.0%)*	*5 (9.8%)*
Total (sum)^c^	1122 (133.9%)		853 (101.8%)	195 (102.1%)	74 (145.1%)

Subcategories are presented in italics.

^a^Loprazolam, flurazepam, flunitrazepam, all prescribed as sleep medication to <1.0% of the patients over two years including the first consultation.

^b^Alprazolam, trazodone, clonazepam, olanzapine, notriptyline, ketotifen, gabapentine, bromazepam, haloperidol, all prescribed as sleep medication to <1.0% of the patients over two years including the first sleep consultation.

^c^Total percentages exceed 100% since patients could receive multiple drugs in two years or as subsequent drugs, and 23 patients (2%) were prescribed >1 drug as initial and/or second drug.

### Characteristics associated with sleep medication prescription

Age was significantly associated with sleep medication prescription, though this differences was small (OR 1.1 [95% CI 1.1 tot. 1.2] per 5 years). No statistically significant differences were found with respect to sex and concurrent psychological problems and social problems. Female patients were as likely as males to receive a prescription (OR 1.0 [95% CI 0.7 to 1.3]). Insomnia patients with a concurrent psychological problem or social problem were as likely than those patients without such problems to receive a prescription (OR 1.3 [95% CI 0.8 to 2.1]) and (OR 0.9 [95% CI 0.6 to 1.3]) respectively. This remained the same when correcting for age (OR 1.0 [95% CI 0.7 to 1.4], OR 1.4 [95% CI 0.8 to 2.3], OR 0.8 [95% CI 0.6 to 1.2], respectively).

### Post hoc analysis stratified by one or more sleep consultation during follow-up

The group of patients with more than one sleep consultation (*N* = 547), compared with the group of patients with a single consultation (*N* = 542), received more often a prescription for sleep medication (85.0% vs 68.8% respectively: OR 2.6 [95% CI 1.9 to 3.5]) and more often more than one prescription (64.0% vs 27.3%: OR 4.7 [95% CI 3.7 to 6.1]). In addition, more often a non-pharmacological intervention was documented (57.8% vs 38.9%: OR 2.2. [95% CI 1.7 to 2.7]). Of the patients who had various sleep consultations in two years, 82 of these patients (15.0% of 547) did not receive sleep medication. On the other hand, of the patients with a single consultation, 148 patients (27.3% of 542) received more than one prescription.

## Discussion

### Statement of principal findings

Over two years including the first consultation GPs documented a non-pharmacological intervention in about half of the patients who consulted their general practitioner (GP) for sleep disturbance for the first time and prescribed sleep medication to 77.0%. For a fifth of the total study population the non-pharmacological intervention preceded sleep medication prescription or this was the only treatment documented. 64.6% of total study population received a sleep medication prescription in the first consultation. About half of the total study population, had one more sleep consultation during the two years follow-up. Among patients receiving medication (*N* = 838) more than a third received repeat prescriptions of one drug and more than a fifth more than one prescription for various drugs in two years. Short-acting benzodiazepine receptor agonists (BZRAs) were most often prescribed, but more than a third received one or more drugs not recommended by the Dutch insomnia guideline such as other BZRAs or melatonin, or off-label drugs including antidepressants, antihistaminics, antipsychotics, anxiolytics and antiepileptics. Almost 15% received long term BZRA treatment (more than 180 pills) over two years.

### Findings in relation to other studies

In our observational study using real-life routine general practice care data, we found that in only half of the patients any non-pharmacological intervention was documented, while the guideline recommends non-pharmacological treatment independent of the type or duration of insomnia. In a fifth of the total study population, this preceded sleep medication prescription or was the only treatment. Figures on non-pharmacological interventions in general practice are scarce. In a Norwegian survey of the general population, only 9.6% of respondents who had ever used sleep medication reported that they had actually been offered a non-pharmacological intervention [32]. In interview and in survey studies on the approaches of GPs to non-pharmacological interventions, the majority of GPs reported to use it in combination with sleep medication prescription, sometimes in a stepwise approach [23,27]. A minority reported that they provided only non-pharmacological interventions. In two studies, however, some GPs reported that they did not offer non-pharmacological interventions and mainly prescribed sleep medication because they considered nonpharmacological interventions ineffective as patients had usually tried numerous self-help methods before consulting them [25,29]. In our study 473 (43.4%) of the patients seemed to receive a prescription only.

In our study GPs prescribed sleep medication in the first consultation to 64.6% of the patients who consulted them for sleep disturbance for the first time (56.0% a BZRA, 8.6% non-BRZA). 45.8% of the total study population received more than one sleep medication prescription in two years. This in line with a previous study in the Netherlands. Over 2008 and 2009 it was found that about 60% of the patients with an incident sleep disturbance episode was prescribed a BZRA in the first 7 days after diagnosis and around 36% received more than one BZRA prescription within 90 days [30]. While there are differences in methodology (e.g. we considered both BRZA and non-BRZA sleep medication and excluded patients with sleep medication prescribed for traveling and pre-operative), our data confirm that sleep medication prescription and prescription rate at the first consultation remain persistently high.

With respect to the nature of drugs prescribed, our routine general practice care data show that GPs mostly prescribed temazepam, which is explicitly mentioned in the main text of the national guideline [2]. In other countries the prescription of z-drugs is more commonly seen [10,32,33]. Our data also GPs prescribed unrecommended (39.5%) or off-label drugs (12.2%). Off-label drugs were more often prescribed as second and subsequent drug. In a survey study in the general Norwegian population the proportion using unrecommended sleep medication was higher [32]; in their study 7.9% of the patients reported to use sleep medication in the past month; 62.7% of them reported to use z-drugs for sleep, 4.4% benzodiazepines, 17.0% anxiolytics, 14.6% antidepressants and 9.2% antipsychotics.

### Strengths and weaknesses of the study

In the present study we analyzed general practice care data on insomnia management, which means that the GPs were no aware of this study while recording the consultations. The unique combination of prescription data and full-text documentation at consultation level enabled us explore the timing of the prescriptions in relation to non-pharmacological interventions and in relation to documented sleep characteristics such as the presence of a crisis. Furthermore, we could identify the wide range of off-label drugs used as sleep medication and distinguish BZRAs prescribed as sleep medication from BZRAs used for other indications (e.g. anxiety).

Using routine care data also has limitations. A major limitation is that GPs do not document everything that is discussed or done during a consultation. Although we included a broad definition for non-pharmacological interventions, not all advices may have been registered in the EMR, as reflected by a small group of patients (8%) receiving neither a documented non-pharmacological intervention nor sleep medication. Furthermore, our exploration did not encompass diagnostic strategies of insomnia management such as the provision of sleep diaries and referrals, since these are generally not annotated in the free-text.

Second, we based our patient selection on the GP’s registration of sleep disturbances (ICPC code P06, excluding P06.1 OSAS) during the consultation. Although this is, except P06.1 for OSAS, the only ICPC code for consultations regarding sleep, we do not have a full view of the selection steps prior to registering this code and how this affects the prescription rate. For example, not all people with sleep disturbance contact their general practice and it is possible that patients come to the GP when they think they really need sleep medication. Subsequently, it might be the case that the sleep disturbance has already been discussed as part of another problem, but not yet coded as P06, and that P06 is registered when medication is prescribed. Since there is only one ICPC code, we had expected to find more information in the free-text about the sleep characteristics, in order to distinguish insomnia symptoms from insomnia diagnosis and insomnia disorder and other sleep disorders. Although lack of registration does not mean that it was not assessed, certainly the diagnostics should be better documented. Even with this in mind, however, there are still many patients who get a prescription when the treatment of first choice is non-pharmacological for all.

Thirdly, based on our research, we are limited in our conclusions about non-pharmacological treatment as we did not distinguish between the different non-pharmacological interventions. Some types of non-pharmacological interventions are known to be more effective than others, and for those patients who have insomnia disorder most of the documented non-pharmacological interventions are not considered as evidence-based interventions. Adding this information could have increased the quality of our exploration of insomnia management. This was, however, not one of our primary research questions and it was often not clearly described in the free-text.

Fourthly, we started our investigation directly after publishing of the guideline. On the one hand that might be the moment GPs pay extra attention to insomnia management due to the publicity involved or the other hand it is known that it will take a while to implement new recommendations. This guideline differed from the previous ones in only a few points. Firstly, recommendations for insomnia of three weeks or longer now included recommendations on behavioral approach and advises on exercise. Secondly, the rationale for limiting the prescription of long-acting benzodiazepine such as diazepam changed from ‘prescribe only if you also want to achieve sedation or anxiolysis during the day‘ into ‘although the rapid onset of action makes it suitable for use in insomnia, its long half-life poses a risk of accumulation with repeated use’.

Lastly, our data present insomnia care for a large group of patients in an urban area, which might not be generalizable to general practices and patients in rural areas of the Netherlands. Furthermore, we cannot draw conclusions about insomnia care to children and the eldest elderly. It would be useful to include these groups in future studies.

### Meaning of the study: possible mechanisms and implications for clinicians or policy makers

Despite guidelines advocating non-pharmacological treatment, insomnia management in general practice is characterized by frequent and prompt sleep medication prescription. Some patients receive various drugs, off-label drugs, or long term BRZA prescriptions and medication without follow up consultations. This contributes to the maintenance of a group of long-term sleep medication users. Our findings stress, on the one hand, that an extra effort is needed to ensure that guideline recommendations regarding insomnia treatment are fully implemented by GPs. On the other hand, deviations from guideline recommendations might reflect unmet needs of patients and GPs such as the limited availability of effective non-pharmacological treatments and effective and safe sleep medication. Policymakers can play a major role in this by organizing a cohesive structure for all facets of insomnia management (i.e. diagnosis, treatment, evaluation) which includes collaborations of general practitioners, specialized nurses, pharmacies and secondary care. Additionally, future research can address the questions arising in clinical practice on which the guidelines have no answer such as to find out in which patients (adjuvant) use of sleep medication is appropriate and which drugs are prescribed best to patients with persisting sleep disturbance when short term short-acting BRZAs are not sufficiently effective.
